# Post-transcriptional control by bacteriophage T4: mRNA decay and inhibition of translation initiation

**DOI:** 10.1186/1743-422X-7-360

**Published:** 2010-12-03

**Authors:** Marc Uzan, Eric S Miller

**Affiliations:** 1 Acides Nucléiques et Biophotonique, FRE 3207 CNRS-Université Pierre & Marie Curie, 4 Place Jussieu, 75252 PARIS cedex 05, France; 2 Department of Microbiology, North Carolina State University, Raleigh, 27695-7615, NC, USA

## Abstract

Over 50 years of biological research with bacteriophage T4 includes notable discoveries in post-transcriptional control, including the genetic code, mRNA, and tRNA; the very foundations of molecular biology. In this review we compile the past 10 - 15 year literature on RNA-protein interactions with T4 and some of its related phages, with particular focus on advances in mRNA decay and processing, and on translational repression. Binding of T4 proteins RegB, RegA, gp32 and gp43 to their cognate target RNAs has been characterized. For several of these, further study is needed for an atomic-level perspective, where resolved structures of RNA-protein complexes are awaiting investigation. Other features of post-transcriptional control are also summarized. These include: RNA structure at translation initiation regions that either inhibit or promote translation initiation; programmed translational bypassing, where T4 orchestrates ribosome bypass of a 50 nucleotide mRNA sequence; phage exclusion systems that involve T4-mediated activation of a latent endoribonuclease (PrrC) and cofactor-assisted activation of EF-Tu proteolysis (Gol-Lit); and potentially important findings on ADP-ribosylation (by Alt and Mod enzymes) of ribosome-associated proteins that might broadly impact protein synthesis in the infected cell. Many of these problems can continue to be addressed with T4, whereas the growing database of T4-related phage genome sequences provides new resources and potentially new phage-host systems to extend the work into a broader biological, evolutionary context.

## Introduction

The temporal ordering of bacteriophage T4 development is assured, in great part, by the cascade activation of three different classes of promoters (see [1,2] in this series). However, control of phage development is also exercised at the post-transcriptional level, in particular by mechanisms of mRNA destabilization and translation inhibition [see earlier reviews [3-6]]. In this review we detail advances in understanding these processes, and summarize some of the other posttranscriptional processes that occur in T4-infected cells.

## Posttranscriptional control by mRNA decay

### Endoribonuclease RegB and its role in inactivating phage early mRNAs

The end of the early period, 5 minutes after infection at 30°C, is marked by a strong decline in the synthesis of many early proteins. This inhibition is due to the abrupt shut-down of the early promoters by a mechanism that is not completely understood [7,8]. In addition, the phage-encoded RegB endoribonuclease (T4 *regB *gene) functionally inactivates many early transcripts and expedites their degradation. As described below, this role of RegB is accomplished in part, with the cooperation of the host endoribonucleases RNase E and RNase G and the T4 polynucleotide kinase, PNK.

The T4 RegB RNase exhibits unique properties. It generates cuts in the middle of GGAG/U sequences located in the intergenic regions of early genes, mostly in translation initiation regions. In fact, the GGAG motif is one of the most frequent Shine-Dalgarno sequences encountered in T4. Some efficient RegB cuts have also been detected at GGAG/U within coding sequences. RegB cleavages can be detected very soon after infection, earlier than 45 seconds at 30°C [5,9-14].

The RegB endonuclease requires a co-factor to act efficiently. When assayed *in vitro*, RegB activity is extremely low but can be stimulated up to 100-fold by the ribosomal protein S1, depending on the RNA substrate [9,15,16].

#### Functional inactivation of mRNA by RegB

The consequence of RegB cleavage within translation initiation regions is the functional inactivation of the transcripts. The synthesis of a number of early proteins starts immediately after infection and reaches a maximum in four minutes before declining abruptly thereafter. In *regB *mutant infections, several of these early proteins continue to be synthesized for a longer time, resulting in twice the accumulation as compared to when RegB is functional. The abrupt arrest of synthesis of these proteins at ~4 min postinfection with wild-type phage results both from the sudden inhibition of early transcription and the functional inactivation of mRNA targets by RegB. However, in addition to down-regulating the translation of many early T4 genes RegB-mediated mRNA processing stimulates the synthesis of a few middle proteins, such as the phage-induced DNA polymerase, encoded by T4 gene *43 *[11,12].

#### RegB accelerates early mRNA breakdown

RegB accelerates the degradation of most early, but not middle or late mRNAs. Indeed, bulk early mRNA is stabilized about 3-fold in a *regB *mutant compared to wild-type infection. After ~3 min post-infection, mRNAs decay with a constant half-life of about 8 minutes for the remainder of the growth period at 30°C, irrespective of the presence or the absence of a functional RegB nuclease [11]. The host RNase E plays an important role in T4 mRNA degradation throughout phage development [17]. Total T4 RNA synthesized during the first two minutes of infection of the temperature-sensitive *rne *host mutant is stabilized 3-fold at non-permissive temperatures. When both genes, *regB *and *rne*, are mutationally inactivated, bulk early T4 mRNA is stabilized 8 to 10-fold (half-life of 50 min at 43°C), showing that both T4 RegB and host RNase E endonucleases are major actors in T4 early mRNA turnover (B. Sanson & M. Uzan, unpublished results).

RegB could accelerate mRNA decay by increasing the number of entry sites for one or the other of the two host 3' exoribonucleases, RNase II and RNase R, which can attack the mRNA from the 3'-phosphate terminus left after RegB cleavage. An alternative pathway was suggested by the finding that some endonucleolytic cleavages within A-rich sequences depend upon RegB primary cuts a short distance upstream. This was interpreted as meaning that RegB triggers a degradation pathway that involves a cascade of endonucleolytic cuts in the 5' to 3' orientation [12]. The host endoribonucleases, RNase G and RNase E, are responsible for cutting at secondary sites, with RNase G playing a major role [14]. This finding appeared paradoxical since these two endonucleases have a marked preference for RNA substrates bearing a monophosphate at their 5' extremities [18-20], while RegB produces 5'-hydroxyl RNA termini. Therefore, we suspected that T4 infection induced an activity able to phosphorylate the 5'-OH left by RegB, and the best candidate for filling this function is the phage-encoded 5' polynucleotide kinase/3' phosphatase (PNK). This enzyme catalyzes both the phosphorylation of 5'-hydroxyl polynucleotide termini and the hydrolysis of 3'-phosphomonoesters and 2':3'-cyclic phosphodiesters. Indeed, Durand *et al*. (2008; unpublished data) showed that the secondary cleavages are abolished in an infection with a phage that carries a deletion of the *pseT *gene, encoding PNK. In addition, many cleavages detected over a distance of 200 nucleotides downstream of the initial RegB cut (mostly generated by RNase E and a few by RNase G), disappear or are strongly weakened in the PNK mutant infection. The availability of a mutant affected only in the phosphatase activity (*pseT1*) made it possible to show that the phosphatase activity of PNK also contributes to mRNA destabilization from the 3' terminus. This presumably occurs through the conversion of 3'-phosphate into 3'-hydroxyl termini, making RNAs better substrates for polynucleotide phosphorylase, the only host 3' exoribonuclease that requires a 3'-hydroxyl terminus to act efficiently. The total inactivation of PNK increases the stability of some RegB-processed transcripts (Durand *et al*. 2008, unpublished data). Thus, both the kinase and phosphatase activities of PNK control the degradation of some RegB-processed transcripts from the 5' and the 3' extremities, respectively. This shows that the status of the 5' and 3' RNA extremities plays a major role in mRNA degradation (see also [21]). This was the first time a direct role was ascribed to T4 PNK in the utilization of phage mRNAs. In bacteriophage T4, as in other phages and bacteria where this enzyme is found, PNK is involved in tRNA repair, together with the RNA ligase, in response to cleavage catalyzed by host enzymes [22,23] (and see below). Durand's finding should prompt one to consider that, in addition to a role in RNA repair, prokaryotic PNKs might participate in the regulation of mRNA degradation.

The data presented above show that RNase G, a paralogue of RNase E in *E. coli*, participates in the processing and decay of several phage transcripts [14] (Durand *et al*. 2008, unpublished data). Nevertheless, it seems clear that it does not have the same general effect on phage mRNA as RNase E. The plating efficiency of T4 is reduced only by 30% on a strain deficient in RNase G (*rng*::Tn5) relative to a wild-type strain (Durand *et al*. 2008, unpublished data).

#### The RegB/S1 target site

It has been obvious since the initial discovery of RegB activity that not all intergenic GGAG sequences are cleaved by this RNase [13,24], suggesting that the motif is necessary but not sufficient for cleavage. RNA secondary structure protects against cleavage and several phage mRNAs that carry an intergenic GGAG/U motif are resistant to the nuclease, including a few early, most middle and all late transcripts [11]. These GGAG-containing mRNAs are not substrates of the enzyme either *in vitro *or *in vivo *[11].

A SELEX (systematic evolution of ligands by exponential enrichment; [25]) experiment, based on the selection of RNA molecules cleaved by RegB in the presence of the ribosomal protein S1, led to the selection of RNA molecules that all contained the GGAG tetranucleotide [26] and no other conserved sequence or structural motif. However, in most cases, the GGAG sequence was found in the 5' portion of the randomized region, suggesting that the nucleotide composition 3' to this conserved motif plays a role. More recently, by using classical molecular genetic techniques, Durand *et al*. [9] showed that this was indeed the case. The strong intergenic RegB cleavage sites share the following consensus: GG*AGRAYARAA, where R is a purine (often an A, leading to an A-rich sequence 3' to the very conserved GGAG motif) and Y a pyrimidine (the star indicates the site of cleavage) [9]. This unusually long nuclease recognition motif is reminiscent of cleavage sites for some mammalian endoribonucleases that function with auxiliary factors. One possible model assumes that the auxiliary factors bind the long nucleotide sequence and recruit the endonuclease [27]. Durand et al. [9] provided evidence that RegB alone recognizes the trinucleotide GGA, which it cleaves very inefficiently, irrespective of its nucleotide sequence context, and that stimulation of the cleavage activity by S1 depends on the base composition immediately 3' to -GGA-.

#### RegB catalysis and structure

The bacteriophage T4 RegB endoribonuclease is a basic, 153-residue protein. Although its amino acid sequence is unrelated to any other known RNase, it was shown to be a cyclizing ribonuclease of the Barnase family, producing 5'-hydroxyl and cyclic 2',3'-phosphodiester termini, with two histidines (in positions 48 and 68) as potent catalytic residues [28].

**Figure 1:**
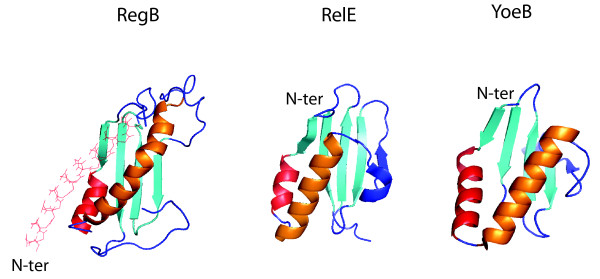
**NMR structures of RegB, RelE and YoeB endoribonucleases**. The structures of RegB [29], RelE [144] and YoeB [145] are shown. The first α-helix of RegB, absent in the two other endoncleases, is drawn in pale orange. The two conserved α-helices are in red and orange and the conserved four-stranded β-sheet is in cyan.

NMR was used to solve the structure of RegB and to map its interactions with two RNA substrates. Despite the absence of any sequence homology and a different organization of the active site residues, RegB shares structural similarities with two *E. coli *ribonucleases of the toxin/antitoxin family: YoeB and RelE [29]. YoeB and RelE are involved in the inactivation of mRNA translated under nutritional stress conditions [30,31]. Interestingly, like RegB, RelE, and in some cases YoeB recognize triplets on mRNAs, which they cleave between the second and third nucleotides. It has been proposed that RegB, RelE and YoeB are members of a newly recognized structural and functional family of ribonucleases specialized in mRNA inactivation within the ribosome [29] (Figure 1).

#### How does S1 activate the RegB cleavage reaction?

The *E. coli *S1 ribosomal protein is an RNA-binding protein required for the translation of virtually all the cellular mRNAs [32]. It contains six homologous regions, each of about 70 amino acids, called S1 modules (or domains) connected by short linkers. S1 binds to ribosomes through its two N-terminal domains (modules 1-2) while mRNAs interact with the C-terminal domain made of the four other modules (3-4-5-6) [33]. S1-like modules are found in many proteins involved in the metabolism of RNA throughout evolution. The structure of these modules, (based on studies of the *E. coli *S1 protein itself as well as RNase E and PNPase), are predicted to belong to the OB-fold family [34-38].

The modules required in RegB activation have been identified. The C-terminal domain of S1 (including modules 3-4-5-6) stimulates the RegB reaction to the same extent as the full-length protein. Depending on the substrate, domain 6 can be removed without affecting the efficacy of the reaction. The smallest domain combination able to stimulate the cleavage reaction significantly is the bi-module 4-5 [9,39]. Interestingly, small angle X-ray scattering studies performed on the tri-module 3-4-5 showed that the two adjacent domains 4 and 5 are tightly associated, forming a rigid rod, while domain 3 has no or only a weak interaction with the others. This suggests that the S1 domains 4 and 5 cooperate to form an RNA binding surface able to interact with the nucleotides of RegB target sites. Module 3 could help stabilize the interaction with the RNA [34].

The 3' A-rich sequence that characterizes strong RegB sites (see above) plays a role in the mechanism of stimulation by S1. Indeed, directed mutagenesis experiments showed that the stimulation of RegB cleavage by S1 depends on nucleotides immediately 3' to the totally conserved GGA triplet. The closer the sequence is to the consensus shown above, the greater the stimulation by S1 [9]. The affinity of S1 for the A-rich sequence is not better than for any other RNA sequence (S. Durand and M. Uzan, unpublished data); suggesting that the function of this sequence is not simply to recruit S1 locally. Rather, specific interactions of S1 with the conserved sequence might make the G-A covalent bond more accessible to RegB. In support of this view, RegB alone (without S1) is able to perform efficient and specific cleavage in a small RNA carrying the GGAG sequence, provided the GGA triplet is unpaired and the fourth G nucleotide of the motif is partly constrained [15]. The RegB protein shows very weak affinity for its substrates [26,28] and in fact, no RegB-RNA complex can be visualized by gel shift experiments. However, in the presence of S1, RegB-RNA-S1 ternary complexes can form, suggesting that the first step in the S1 activation pathway involves S1 interaction with the RNA (S. Durand and M. Uzan, unpublished observations). Taken together, these observations suggest that through its interaction with the A-rich sequence 3' to the cleavage site, the S1 protein promotes a local constraint on the RNA, facilitating the association or reactivity of RegB.

As RegB is easily inhibited by RNA secondary structures, one possibility was that S1 stimulates RegB through its RNA unwinding ability [40,41]. However, Lebars *et al*. [15] provided evidence that does not support this hypothesis.

Whether S1 participates in the RegB reaction as a free protein or in association with the ribosome or other partners *in vivo *remains to be determined. However, the structural and mechanistic analogy of RegB to the two *E. coli *RNase toxins, YoeB and RelE [29], which depend on translating ribosomes for activity [30], and the efficiency of RegB cleavage *in vivo *very shortly after infection [13], favor the likelihood of ribosomes participating in RegB processing of mRNAs *in vivo*.

#### Regulation and distribution of the regB gene

The *regB *gene is transcribed from a typical early promoter that is turned off two to three minutes after infection. The *regB *gene is also regulated at the post-transcriptional level, suggesting that the production of this nuclease must be tightly regulated. Indeed, RegB efficiently cleaves its own transcript in the SD sequence, indicating that RegB controls its own synthesis. Three other cleavages of weaker efficiency occur in the *regB *coding sequence, which probably contribute to *regB *mRNA breakdown [10].

Despite the fact that the RegB nuclease seems dispensable for T4 growth, the *regB *gene is widely distributed among T4-related phages. The *regB *sequence was determined from 35 different T4-related phages. Thirty-two of these showed striking sequence conservation, while three other sequences (from RB69, TuIa and RB49) diverged significantly. As in T4, the SD sequence of these *regB *genes is GGAG, with only one case (RB49) of GGAU. When experimentally tested, this sequence was always found to be cleaved by RegB *in vivo*, suggesting that translational auto-control of *regB *is conserved in T4-related phages [42].

Mutants of *regB *are viable on laboratory *E. coli *strains, although their plaques are slightly smaller in minimal medium than those of the wild-type phage. Also, T4 *regB *mutants form minute plaques on the hospital *E. coli *strain CTr5x, with a plating efficiency of one third that on classical laboratory strains (M. Uzan, unpublished data).

#### What is the role of RegB in T4 development?

Early transcripts are synthesized in abundance immediately after infection, reflecting the exceptional strength of most T4 early promoters. In fact, effective promoter competition for RNA polymerase can be considered one of the first mechanisms leading to shut-off of host gene transcription. Abundant and stable phage early transcripts would compete for translation with the subsequently made middle and late transcripts. Therefore, a specific mechanism leading to early mRNA inactivation and increased rate of degradation should free the translation apparatus more rapidly and facilitate the transition between early and later phases of T4 gene expression [5]. Functional mRNA endonucleolytic inactivation is certainly a faster means to arrest ongoing translation and rapidly re-orient gene expression in response to changes in growth conditions or the stage of development. In this regard, it is striking that the two toxin endoribonucleases, RelE and YoeB, to which RegB shows strong structural similarities (Figure 1) [29], also allow swift inactivation of translated mRNAs in response to nutritional stress.

The finding that RegB shares structural and functional similarities with other toxin RNases that have antitoxin partners raises the possibility that an anti-RegB partner might be encoded by T4. On the other hand, RegB might not require an antitoxin to block its activity since its *in vivo *targets disappear through mRNA decay shortly after it acts in the infected cell.

### T4 Dmd and E. coli RNase LS antagonism

#### T4 Dmd controls the stability of middle and late mRNAs

The T4 early *dmd *gene (*d*iscrimination of *m*essages for *d*egradation) encodes a protein that controls middle and late mRNA stability. Indeed, an amber mutation in *dmd *leads to strong inhibition of phage development. Protein synthesis is normal until the beginning of the middle period and collapses thereafter. A number of endonucleolytic cleavages can be detected in middle and late transcripts, which are not present in wild-type phage infection. Consistent with this observation, the accumulation of these RNA species drops dramatically and the chemical and functional half-lives of several middle and late transcripts were shown to be shortened [43-46]. The host RNA chaperone, Hfq, seems to enhance the deleterious effect of the *dmd *mutation [47]. These data strongly suggest that the arrest of protein synthesis in T4 *dmd *mutants is the consequence of mRNA destabilization and that the function of the Dmd protein is to inhibit an endoribonuclease that targets middle and late transcripts.

The endoribonuclease responsible for middle and late mRNA destabilization in the *dmd *mutant is of host origin as shown by the fact that a late mRNA (*soc*) produced from a plasmid in uninfected bacteria undergoes the same cleavages as those observed after infection by a *dmd *mutant phage [43,48]. Yonesaki's group further showed that this RNase activity depends on a new endonuclease, RNase LS, for *l*ate gene *s*ilencing in T4. Several *E. coli *mutants able to support the growth of a *dmd *mutant phage were isolated, among which, two very efficiently reversed the *dmd *phenotype. Both mutations were mapped within the ORF *yfjN*, which was renamed *rnlA *[44,45,48].

#### Biochemical characterization of RNase LS

Purified his-tagged RnlA protein cleaves the late *soc *transcript *in vitro *at only one site among the three usually observed *in vivo *after infection with *dmd *mutant phage. This cleavage is inhibited by purified Dmd protein [49]. Thus, RnlA has an RNase activity that responds directly to Dmd. Whether RnlA has targets in other T4 mRNAs remains to be determined.

Biochemical experiments showed that RNase LS activity is associated with a large complex whose MW was estimated to be more than 1,000 kDa. More than 10 proteins participate in the complex. Two of them were identified: RnlA and triose phosphate isomerase. The latter is present in stoichiometric amounts relative to RnlA and binds very tightly to it [45,49]. Interestingly, a mutation in the gene for triose phosphate isomerase is able to partially allow the growth of a T4 *dmd *mutant, suggesting that RnlA and triose phosphate isomerase functionally interact. It is unclear whether RNase LS carries only one RNase activity (presumably that of the RnlA protein) or more, and if the activity of RnlA is modulated by other components of the complex.

The multi-protein complex that constitutes RNase LS is not simply a modification of the host degradosome to contain the RnlA protein during T4 infection, since the *dmd *phenotype is not reversed in infection of an RNase E host mutant (*rne*Δ131) unable to assemble the degradosome [48].

#### The specificity of RNase LS and coupling with translation

The specificity and mode of action of RNase LS are not yet understood. Most of the ~30 cleavages analyzed in various middle and late transcripts occur 3' to a pyrimidine in single-stranded RNA. Also, nucleotides 3' to the cleavage site might play a role. Apart from these observations, no sequence or structural motif seems to be shared by the RNase LS target sites [43,44,50,51].

The presence of ribosomes loaded on the mRNA seems to be required for some RNase LS sites to be efficiently cut. The ribosomes may be either translating or pausing at a nonsense codon. In the later case, new cleavage sites by RNase LS appear at some distance (20-25 nucleotides) downstream of the stop codon [44,48,51]. It has been suggested that ribosomes act through their RNA unwinding property, maintaining the RNA in a locally single-stranded conformation. In the absence of translation, a number of potential RNase LS sites would be masked by secondary structure [51]. Whether this is the only role of the ribosome in RNase LS activation is an open question.

#### The role of RNase LS in E. coli

A mutation in the *E. coli rnlA *gene, whether a point mutation or an insertion, leads to reduction in the size of colonies on minimum medium, but has no effect on growth in rich medium. Growth of *rnlA *mutants is however dramatically affected in rich medium supplemented with high sodium chloride concentrations, thus providing a phenotype for *rnlA *mutants. RNA is stabilized by 30% on average in an *rnlA *mutant. RNase LS was shown to participate in the degradation of specific mRNAs as reflected by the prolonged functional lifetime of several mRNAs in the *rnlA *mutant. The *rpsO*, *bla *and *cya *mRNAs are stabilized 2 to 3-fold, in the *rnlA *mutant, while other transcripts are unaffected. The greater stability of *cya *mRNA (adenylate cyclase) in an *rnlA *mutant might indirectly account for the sensitivity of *rnlA *cells to NaCl [45,52]. In addition to moderately controlling the decay of some bacterial transcripts, it is possible that the first function of RNase LS is host defense against phage propagation and Dmd is a phage response to overcome the host defense.

### Other activities implicated in RNA decay during T4 infection

The *E. coli *poly(A) polymerase (PAP), encoded by the *pcnB *gene, adds poly(A) tails to the 3' ends of *E. coli *mRNAs and contributes to the destabilization of transcripts [53]. T4 mRNAs are probably not polyadenylated. Indeed, it has been found that after infection with the closely related bacteriophage T2, host poly(A) polymerase activity is inhibited [54]. Also, no poly(A) extension could be detected at the 3' end of the *soc *and *uvsY *transcripts after infection with T4 [55], suggesting that bacteriophage T4 infection also leads to PAP inhibition. This could, for example, occur through ADP-ribosylation of the protein.

Growth of bacteriophage T4 on an *E. coli *strain carrying the *rne*Δ131 mutation, which is unable to assemble the RNA degradosome, is unchanged relative to infection of a wild-type strain [48] (also, S. Durand and M. Uzan, unpublished data). However, the *rne*Δ131 mutation has no effect on the growth of *E. coli *either, despite affecting the stability of several individual transcripts [56-59]. Therefore, the question of whether the degradosome plays a role in the turnover of some T4 mRNAs or is modified after infection remains open. Similarly, whether the host RNA pyrophosphohydrolase, RppH [21,60] is implicated in T4 mRNA turnover has not yet been determined.

Infection with bacteriophage T4 expedites host mRNA degradation. The two long-lived *E. coli *mRNAs, *lpp *and *ompA*, are dramatically destabilized after infection with T4. The host endonucleases, RNases E and G, are responsible for this increased rate of degradation [61]. Phage-induced host mRNA destabilization requires the degradosome. Indeed, the *lpp *mRNA is not destabilized after infection of a strain that carries a nonsense mutation in the middle of the *E. coli rne *gene (encoding RNase E), leading to a protein unable to assemble the degradosome. A viral factor is also involved, since a phage carrying the Δ*tk2 *deletion that removes an 11.3 kbp region of the T4 genome, from the *tk *gene to ORF *nrdC*.2, loses the ability to destabilize host transcripts. The gene implicated has not yet been identified [61]. There is certainly an advantage for a virulent phage to accelerate host mRNA degradation immediately after infection, as this provides ribonucleotides for nucleic acid synthesis, frees the translation apparatus for viral mRNAs, and facilitates the transition from host to phage gene expression.

A list of the several endoribonucleases and other enzymes involved in mRNA degradation and modification during T4 infection is presented in Table 1.

**Table 1:** Enzymes involved in mRNA degradation and modification during T4 infection.

Enzyme	Origin	Reaction catalyzed. Main properties	Role in T4 development
RNase E	*E. coli*	Endonuclease. Produces 5'-P termini. Activated by 5'-monophosphorylated RNA. Scaffold of the degradosome	Major role in mRNA degradation throughout the phage developmental cycle.
RNase G	*E. coli*	Endonuclease. Produces 5'-P termini. Activated by 5'- monophosphorylated RNA.	Cuts in the 5' regions of some early RegB processed transcripts.
RegB	T4	Sequence-specific endonuclease. Produces 5'-OH termini. Requires S1 r-protein as co-factor	Inactivates early transcripts by cleaving in Shine-Dalgarno sequences. Expedites early mRNA degradation.
RNase LS	*E. coli*	Endonuclease. Its activity depends on *rnlA *and *rnlB *loci. Associated in a multiprotein compex.	Cleaves within T4 middle and late transcripts and expedites their degradation.
RNase II RNase R Polynucleotide phosphorylase	*E. coli*	3'-5' exonucleases. PNPase requires 3'-OH termini; the other two are indifferent to the nature of the 3' terminus.	Degrade mRNAs. The relative contribution of each RNase has not been determined.
PrrC	*E. coli*	tRNA^lys ^anticodon nuclease. Normally silent in *E. coli *but activated by the T4-encoded Stp polypeptide.	Deleterious to T4 propagation if Pnk or Rli1 enzymes are inactivated.
Polynucleotide kinase (PNK)	T4	Phosphorylation of 5'-OH polynucleotide termini. Hydrolysis of 3'-terminal phosphomonoesters and of 2',3'-cyclic phosphodiesters	Counteracts, together with T4 RNA ligase 1, host tRNA anticodon nuclease PrrC. Makes RegB-processed RNA substrates for RNases E and G.
Dmd	T4	An early product that binds the RnlA protein, a member of RNase LS	Antagonist of RNase LS
Poly(A) polymerase	*E. coli*	Addition of poly(A) tails to the 3' end of RNAs	Probably inactivated after T4 infection
RNA pyrophospho-hydrolase (RppH)	*E. coli*	Hydrolysis of a pyrophosphate moiety from the 5'-triphosphorylated primary transcripts.	Not yet investigated

## Inhibition of translation initiation

### RegA translational repression

Inhibition of middle transcription, some 12-15 minutes post-infection at 30°C, is concomitant with the strong activation of late transcription [62]. This is the consequence of competition among sigma factors and changing the promoter specificity of the modified host RNA polymerase. Indeed, transcription initiation at T4 late promoters requires the phage-encoded late σ-factor, gp55, which replaces the major host σ70, and the T4-encoded gp33, which ensures coupling of late transcription with ongoing viral DNA replication [1,62-64]. Superimposed on this transcriptional regulation, the translation of a number of transcripts is inhibited by the RegA translational repressor. This small, 122 amino acid protein competes with the ribosome for binding to the translation initiation regions of approximately 30 mRNAs [65]

#### RegA protein

**Figure 2:**
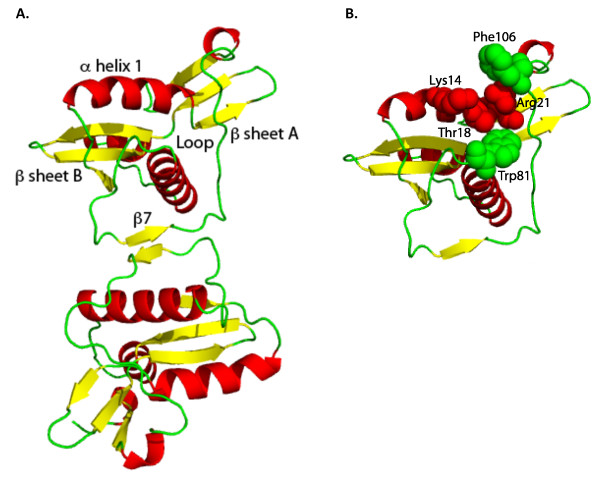
**Crystal structure of T4 RegA**. In panel **A**, the RegA dimer (pymol rendering of PDB 1REG; [66]) is labeled at relevant structures discussed in the text. Panel **B **highlights the likely RNA binding residues in α helix 1 (K14, T18, R21) and loop residue W81. Also shown is the F106 residue that cross-links to bound RNA and is adjacent to the RNA binding region. See Figure 3 for the relative conservation of the labeled amino acids in other RegA proteins. *Adapted from the data of *[66,70,72].

The crystal structure of T4 RegA is a homodimer, with symmetrical pairs of salt bridges between Arg-91 and Glu-68 and pairs of hydrogen bonds between Thr-92 of both subunits [66] (Figure 2). The monomer subunit has an alpha-helical core and two anti-parallel beta sheet regions. Two of the beta strands in the four-stranded beta sheet region B were identified by Kang *et al*. [65] as having amino acid sequences similar to RNP-1 and RNP-2 that are well characterized RNA-binding motifs. In addition, two pairs of lysines, K7-K8 and K41-K42 are in the same position in the proposed RegA RNP-1 domain [66] as they occur in the U1A RNA-binding protein, where they comprise basic "jaws" that straddle the RNA. However, none of the *regA *mutations identified in either T4 or phage RB69 prior to the availability of the RegA structure affected these lysine residues [65]. Structure-guided mutagenesis summarized below also did not implicate the lysines or the RNP-like domains in direct RNA binding by RegA.

Concurrent with the T4 RegA structure determination, E. Spicer's group reported a terminal deletion mutant having residues 1 - 109 that bound RNA with reduced affinity, with 28% of the free energy of binding attributed to the terminal 10% of the protein [67]. It was also shown by proteolytic cleavage of free RegA, and RegA bound to an RNA oligonucleotide (the gene *44 *operator), that conformational change in RegA upon RNA binding affected access to the C-terminal region. The C-terminal region is part of beta sheet region A of RegA [66], appears to be solvent-exposed, and thus potentially could interact with RNA in some manner. However, with the RegA structure available, targeted substitutions in the protein would reveal that specific RNA recognition likely occurs in an entirely different region of the protein.

Structure-guided mutagenesis of RegA was undertaken to evaluate some of these findings and for understanding the specific interactions for RNA binding. Binding stoichiometry of RegA:gene *44 *RNA complexes, gluteraldehyde cross-linking of RegA, and mutagenesis of amino acids in the inter-subunit interface showed that T4 RegA is a dimer in solution (as also revealed in the crystal structure), but binds RNA as monomer [68]. A 1:1 RNA:RegA monomer stoichiometry was independently shown using electrospray ionization mass spectrometry [69]. Mutagenesis of Arg91 again suggested that at least some residues in the C-terminal region are involved in subunit interactions and in RNA recognition [66-68]; Arg91 appears more relevant for RNA binding, whereas Thr92 is more relevant for dimerization. Spicer and colleagues further demonstrated that 19 mutations substituting amino acids in T4 RegA surface residues of both beta structures, including residues similar to the RNP-1 and RNP-2 motifs proposed by Kang *et al*. [66], as well as the two paired lysines, had essentially no affect on RNA binding affinity or on RegA structure [70]. Together with mutations in helix-A, and interpretation of mutations in T4 and RB69 *regA *that were isolated prior to the structure determination [71], a somewhat unique RNA-binding helix-loop groove (or "pocket") of RegA was proposed to provide the primary RNA recognition element for the protein. Modeling of the 78% conserved phage RB69 RegA protein showed that it also likely contains this unique RNA binding structure [72]. Exposed residues on helix-A (i.e., Lys14, Thr18, Arg21) are conserved and substitutions reduce RNA binding substantially. Additionally, a conserved loop Trp81 to Ala81 substitution in both proteins abolishes RNA binding [72]. Phe106, earlier shown to crosslink with bound RNA, is positioned in a loop bordering the other end of the helix and further defines the apparent binding pocket [67,70,72]. Figure 2 summarizes these findings.

**Figure 3:**
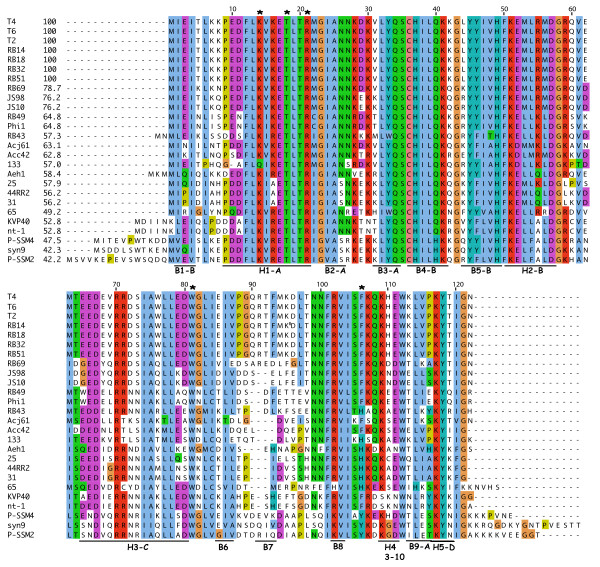
**Aligned RegA proteins of 26 T4-related phages**. *regA *is immediately distal to gene *62 *in the core DNA replication gene cluster of all T4-related genomes sequenced to date. Identity relative to T4 RegA is in column 2, aligned amino acids are shown using ClustalW colors, and dashes are gaps in the alignment. Residues numbered above the sequences reference the T4 protein. Asterisks mark the amino acids cited in the text as involved in RNA binding. At the bottom of the alignment are underlined structural elements of the protein from PDB 1REG[66]. Sequences were obtained from GenBank or the T4-like phage genome browser (http://phage.ggc.edu/).

In summary, biochemical and structural studies of T4 and RB69 RegA have led from inferences of possible motifs in RNA binding to structure guided mutagenesis revealing a unique protein pocket or groove that, in the monomer form, accommodates the many different mRNAs that RegA proteins bind to cause translational repression. The apparent binding domain and exposed amino acids are largely conserved in RegA proteins from diverse phages sequenced to date (Figure 3). As for gp32 and gp43, a RegA-RNA complex has not been structurally resolved and additional analysis of RegA-RNA interactions in the helix-loop groove would be of interest.

#### RegA RNA operators

Early genetic and translational repression assays confirmed that RegA binding sites on mRNA overlap the AUG translation initiation codon, or are located immediately 5' to the AUG, and occluding the site reduces formation of the ternary translation initiation complex; decay of the repressed messages is then enhanced [65]. The lack of clear sequence conservation or secondary structure to define RegA binding sites in the ~30 mRNAs repressed, prompted use of RNA SELEX with T4 RegA to capture high-affinity RNA ligands. This RNA binding site selection was thus performed in the absence of constraints imposed on the sequence by 30S ribosome subunits that bind the same region of mRNA for translation initiation [73]. Emerging from multiple rounds of SELEX was an RNA consensus sequence of 5'-aaAAUUGUUAUGUAA-3' that bound RegA with an apparent Kd of 5 nM (the lower case 5' bases were already present in the starting, non-variable regions of the RNA). The sequence showed no apparent structure using nuclease or base-modifying chemical probes and is consistent with earlier observations that biologically relevant RegA binding sites lack clear RNA secondary structure. Although the T4 RegA SELEX sequence is similar to mRNA sequences repressed by RegA (i.e., T4 gene *rIIB*, AAAAUUAUGUAC; gene *44*, AAAUUAUGAUU; *dexA*, AAAAUUUAAUGUUU), there was no exact match between it and the repressed T4 messages [73]. These findings emphasize that T4 RegA binding sites are A+U rich; include an AUG and a 5' poly(A) tract; lack apparent structure; and in general, illustrate how an RNA binding determinant has evolved for occurring on many different mRNAs where fMet-tRNA and the 30S ribosome subunit also bind.

RNA sequences bound by phage RB69 RegA have also been examined [65,72,74,75]. Translational repression occurs at RNAs from both phages, although binding affinities displayed by the two proteins are different *in vivo *and *in vitro*; a hierarchy of early and middle genes repressed by T4 RegA is also seen with RB69 RegA. For RB69 RegA, the protein protected a region between the gene *44 *and gene *45 *Shine-Dalgarno and AUG, but not the initiator AUG itself [72]. The protein would still compete for the same binding site as the ribosome. Using a stringent but reduced number of selection cycles, RNA SELEX was performed using immobilized RB69 RegA and a variable sequence of 14 bases [75]. The selected RB69 RegA RNAs were predominately 5''AAUAAUAAUAAnA-3', which also did not contain a conserved AUG but were clearly A+U rich. As discussed by Dean *et al*. [75], a stop codon (i.e, UAA) for an upstream gene within the ribosome binding site region of the adjacent downstream gene, may contribute a relevant sequence for RNA recognition by RegA proteins. All of these findings emphasize the range of RegA repression efficiencies at different sites, lack of RNA structure in binding sites, and the variable mRNA sequences to which the protein binds.

### Specific autocontrol of translation: gp32 and gp43

Besides the two general post-transcriptional regulators, RegA and RegB, the T4 DNA unwinding protein, gp32, and the DNA polymerase, gp43, both involved in DNA replication, recombination and repair, autogenously regulate their translation.

#### Control of gene 32 translation and mRNA degradation

Gene 32 encodes a single-stranded DNA binding protein (gp32) essential for replication, recombination and repair of T4 DNA. It appears after a few minutes of infection, reaches a maximum around the 12-14^th ^minute and declines thereafter. In addition to being temporally regulated at the transcriptional level, gp32 inhibits its own translation when the protein accumulates in excess over its primary ligand, single-stranded DNA. This regulation is achieved through binding of gp32 to a pseudoknot RNA structure located 5' in region 67 nucleotides upstream of the gene 32 translation initiation codon. This binding is thought to nucleate cooperative binding through an unstructured A+U-rich sequence (including several UUAA(A) repeats 3' to the pseudoknot) that overlaps the ribosome binding site [3,6,65].

**Figure 4:**
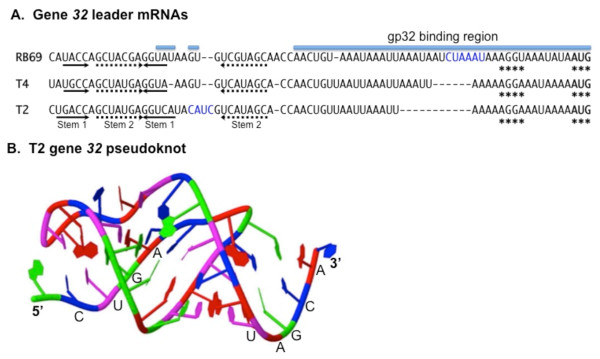
**Gene *32 *translational repression site**. In Panel **A **the leader mRNA for autogenous gp32 binding is shown for RB69, T4 and T2. The important TIR nucleotides are underscored with asterisks, the base-paired regions of the 5' pseudoknot are marked with arrows, and the T4 and RB69 regions bound by gp32 in protection assays are overlined [78]. Short nucleotide insertions in RB69 or T2 relative to T4 are in blue. Dashes (gaps) are inserted for alignment. Panel **B **is a cartoon-ribbon diagram of the T2 gene *32 *pseudoknot diagramed in panel A that was obtained by multidimensional NMR methods [77]. Two A-form coaxially stacked stems are apparent. 5' and 3' terminal nucleotides are labeled. Jmol rendering used database entry 2 tpk. *Figure was derived and adapted primarily from data in *[77,78].

Gp32 is a Zn(II) metalloprotein with three distinct binding domains [76]. To date, the structure of full-length gp32 has not been determined, nor has the protein in complex with RNA been structurally examined. It has been presumed that DNA and RNA are alternative ligands that bind in the same cleft. Although there is substantial study of gp32 interactions with ssDNA, and with proteins of the DNA replication apparatus, few studies have investigated either the RNA pseudoknot in the mRNA autoregulatory site or the molecular details of gp32-RNA interactions. NMR analysis of the phage T2 gene 32 pseudoknot revealed two A-form helices coaxially stacked, with two loops separating the two helical structures [77] (Figure 4). A related translational regulatory structure is present in gene 32 leader mRNA of the phylogenetically related T4-type phage RB69 [78]. In this case, sequence alignment, chemical- and RNase-sensitivity, and gp32-RNA footprinting revealed mRNA operator similarities and differences that explain overlapping yet distinct RNA-binding properties by the two gene 32 proteins [78]. However, the T4-type coliphage RB49 genome sequence revealed no conserved pseudoknot or an A+U-rich sequence near the predicted ribosome binding site of its gene *32 *mRNA [79]. More thorough study of translational autocontrol by gp32 in diverse T4-related phages is needed. To date, the T4-type phage gene *32 *RNA pseudoknot may still be the only viral example of this structure used in autoregulation of translation. The various biological roles of viral RNA pseudoknots was well reviewed by Brierley *et al*. [80].

The gene *32 *transcripts are more stable than any other T4 mRNAs. A half-life of 15 minutes was measured at 30°C and, under derepression conditions (in a T4 gene 32 mutant infection unable to achieve translation repression), the half-life can reach 30 minutes [81,82], indicating that translation of the gene *32 *mRNA positively affects its stability. All the gene 32 mRNA species are processed by RNase E, 71 nucleotides upstream of the translation initiation codon of the gene [83,84]. In addition to the cleavage at -71, two other major cleavages were identified, one far upstream in the polycistronic transcripts (-1340) and the other at the end of the coding sequence of gene *32 *(+831) [85,86]. The conservation of all three RNase E processing sites in 5 different T4-related phages, in spite of significant changes in the organization of the upstream regions, suggests that these cleavages play an important role in controlling expression of gene *32 *and/or its upstream genes [86]. The new 3' ends created by RNase E processing are potential entry sites for the host 3'-5' exoribonucleases. In fact, portions of the transcript upstream of the -71 and -1340 cleavage sites were shown to be rapidly degraded [84,85].

The RNase E cleavage at +831 has no consequences on the functional decay of the gene 32 mRNA, while it affects the chemical decay [17]. It is noteworthy that this RNase E site is very close to the translation termination codon of gene 32. The *E. coli *ribosomal protein S15, encoded by the *rpsO *gene, autogenously regulates its own translation. The *rpsO *transcript carries a pseudoknot in its translational operator [87], like the T4 *32 *mRNA. Also, a strong RNase E cleavage site, involved in *rpsO *mRNA decay, lies at the end of the structural gene, in close proximity of the translation termination codon. Interestingly, ribosomes were shown to inhibit this distal RNase E cleavage [88]. On this basis, it is tempting to suggest that a ribosome that reaches the end of gene 32 transcript would hinder the accessibility of the distal RNase E site to RNase E. Thus, gene 32 transcripts that undergo RNase E processing at this site might be only those that have been already translationally inactivated, e.g., under repression conditions (excess of gp32 over single stranded DNA). This situation would promote rapid elimination of the untranslated gene 32 transcripts.

#### Autocontrol of gene 43 translation

Like gp32, T4 DNA polymerase (gp43) is an autoregulatory translational repressor protein; it binds an RNA operator sequence that includes a hairpin about 40 bases upstream of its translation initiation codon and sequence that overlaps the ribosome binding site [89]. Most T4 gene *43 *transcripts are synthesized early during infection and have a half-life of approximately 3 minutes, yet it is these transcripts on which the polymerase exerts translational repression when not engaged in DNA replication [65].

#### gp43 RNA-binding determinants

The structure of the closely related gp43 DNA polymerase of phage RB69 serves as an excellent model for α DNA polymerases that are conserved across phylogenetic domains [90,91]. Due to the availability of the RB69 gp43 structure, more recent RNA binding studies have been conducted using this protein and its RNA operator.

RB69 operator RNA chemically crosslinks with gp43 in the DNA binding "palm" domain, but other sites and residues protected from protease when the protein is bound to specific RNA were distributed across domains of the polymerase. These numerous affects were attributable to either direct interactions, or conformational changes induced by RNA binding [92]. As for the gp32-RNA interactions, full appreciation of the contacts and conformational changes during binding of gp43 to its specific RNA target will require solution or crystal structure of gp43-RNA complexes.

#### Gene 43 mRNA autoregulatory site

The gene *43 *RNA operator includes an upstream hairpin, but there is no evidence that it forms a pseudoknot structure like that of the gene *32 *binding site. While the T4 hairpin-loop operator is 18 bases and that of RB69 is 16 bases, the top 10 bases are identical, including nucleotides in the loop [93]. The -UAAC- loop sequence of the T4 & RB69 operators were also the predominant bases selected in the first RNA SELEX experiment that used gp43 for RNA binding site characterization [25]; it will be interesting to see whether any phage gp43 proteins closely related to the T4 protein have the SELEX major variant loop sequence (-CAAC-) in their native, autoregulatory RNA hairpins. Phage RB49 contains -UAAA- in its RNA loop, and various repression and RNA-protein interaction assays point to the 3' AC and AA loop bases as especially relevant for binding by these three phage proteins; however, some T4-related phages encode gp43 DNA polymerases that do not autoregulate translation [92-94].

## Other T4 post-transcriptional control systems

### RNA structure at translation initiation regions

RNA structure influences translation initiation of T4 mRNAs, especially as they target protein binding in translational repression (i.e., gp32 and gp43 above; [65,95]). In addition, some T4 mRNAs form intramolecular RNA structures that directly contribute to translation initiation efficiency of the respective mRNAs. Only a few advances have been made in the last decade on these *cis*-acting RNAs, which are briefly summarized here. We should note that no riboswitch system [96,97] or small, *trans*-acting regulatory RNA has been functionally characterized from T4; maybe some of the genome sequences of T4-related phages will suggest good candidates for these types of RNAs. Two small RNAs, RNAC and RNAD, are transcribed from the T4 tRNA region, but their biological roles are unknown [95].

**Figure 5:**
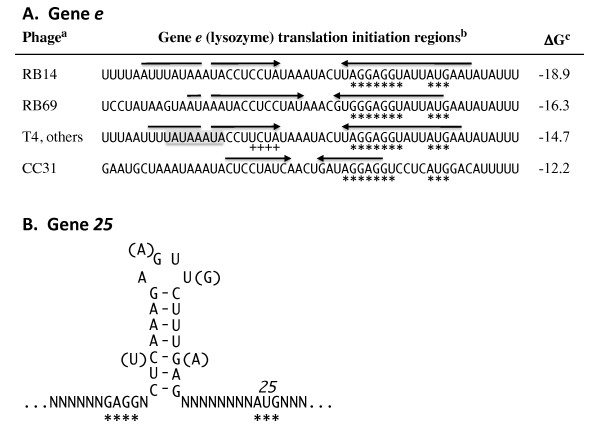
**RNA structures affecting translation at T4-related phage TIRs**. Panel **A **shows stem-loop regions that inhibit translation from early transcripts containing gene *e*. a) Phages are grouped if they have identical TIR regions. Other phages with *e *leaders identical to T4 include: T4T, T2, T6, RB18, RB26, RB32 and RB51. Those having gene *e *but no apparent RNA structure in the TIR: Aeh1, 44RR, 25, 31 & FelixO1. Phages examined but with no apparent gene *e*: RB16, RB43, RB49, phi-1, syn9, S-PM2, PSSM2, PSSM4, 65, 133, KVP40, nt-1, acj009, acj61. b) Gene *e *TIR nucleotides are marked with asterisks. Arrows mark the stems of the likely structures, which was demonstrated for T4 [98]. T4 bases noted with + are the mapped 5' transcript ends from the upstream late promoter (TATAAATA; shaded). Sequences were obtained from GenBank or the T4-type phage browser at http://phage.ggc.edu/. c) RNA folding and ΔG values were by the method of M. Zuker (http://mfold.rna.albany.edu/). Panel **B **shows the conserved stem at the gene *25 *TIR of approximately 30 T-even related phages. Nucleotides of the TIR are indicated with an asterisk, with less conserved adjacent nucleotides noted with N. *Panel B was derived from the data of *[109].

Examples of inhibitory RNA structures at translation initiation regions include mRNAs encoded by T4 genes *e*, *soc*, *49*, and I-TevI [65]. In each case, the Shine-Dalgarno and/or the AUG start codon are sequestered in an RNA helix that reduces 30S subunit binding in forming the ternary translation initiation complex [98]. The well-documented case for gene *e *(T4 lysozyme) is that early during infection longer transcripts are made that extend into *e *and if translated could potentially lead to premature cell lysis. However, the longer transcripts clearly form the inhibitory RNA structure [98], reducing synthesis of lysozyme 100-fold [99] relative to transcripts lacking RNA structure. Transcripts initiated from either of two T4 late promoters immediately upstream of the ribosome binding site lack the 5' portion of the gene *e *mRNA inhibitory structure and are well translated. Although there is no additional analysis of the *e *mRNA structures, similar leader RNA sequences are predicted from genome sequences of closely related T4-type phages, and each has a T4-type late promoters in upstream region the encodes the 5' strand of the RNA structure. Therefore, early translation of these lysis genes may also be inhibited by intramolecular RNA structures (Figure 5).

The T4 thymidylate synthase gene (*td*) contains an intron, wherein the intron encodes a homing endonuclease, I-TevI [100]. Similar to gene *e*, early and middle period transcripts that extend through the *td *5' exon and into the intron do not yield translated I-TevI because of the tightly regulated, sequestered ribosome binding site [101-103]. Recently, Edgell and colleagues [104] showed that deletion of nucleotides that comprise the late promoter proximal 5' portion of the RNA structure leads to increased levels of I-Tev1 throughout infection that is translated from upstream-initiated early and middle promoters. In the presence of added thymidine, the mutant phage (ΔHP) showed no reduction in T4 viability or burst size attributable to increased translation initiation of I-TevI mRNA. However, a series of phage growth, RT-PCR, and tRNA suppressor assays, led Gibb and Edgell [104] to conclude that tight regulation of I-TevI translation initiation by the RNA secondary structure increases intron splicing. That is, the structure reduces ribosome loading and movement through the intron RNA, thereby promoting structure formation (P6, P6a and P7) in the intron. Loss of translation inhibition disrupts intron RNA folding and splicing, and prevents proper accumulation of thymidylate synthase [104]. Similar structures predicted to cause negative translational regulation in I-TevI RNAs have been identified in T4-related phages, and also in the translation initiation regions of phage I-TevII and I-TevIII homing endonuclease genes [105,106]. Stand-alone homing endonucleases (not located within an intron) also have RNA structures that have been shown (*Aeromonas *phage Aeh1 *mobE*; [106]) or implicated (T4 *segB*; [107]) to reduce translation initiation.

Intramolecular RNA structures in T4 mRNAs also have been shown to improve translation initiation. Of particular note are T4 genes *38 *and *25 *[108]. In these cases, suboptimal, extended spacing between the Shine-Dalgarno and AUG start codon is brought to a functional distance by an RNA secondary structure between the SD and AUG. For gene *38 *mRNA, the spacing of 22 nucleotides is reduced to 5 nucleotides with the structure; for gene *25 *the structure reduces the spacing from 27 to 11 nucleotides [108]. Mutations in the intervening sequence that destabilize the structure reduce translation initiation efficiency. More recently, Malys and Nivinskas [109] used reporter assays of the gene *25 *TIR region fused to *lacZ*, in conjunction with DMS probing of the intervening RNA structure, to confirm the "split" RBS-SD arrangement and its use for effective expression of gene *25*. Phylogenetic evaluations of 38 T4-related phages revealed that the close T-even phages all have the intervening RNA structure in the split TIR configuration, but more distant, non-coliform T4-related phages lack this arrangement (Figure 5). This suggested an evolutionary history for the gene *25 *split TIR, along with the enhancing, intervening RNA structure, where the arrangement arose after the close T-even phages diverged from other members of the phage group [109].

### T4 exclusion and the mechanism of bacterial PrrC anticodon nuclease

T4 mutants defective in polynucleotide kinase (Pnk) or RNA ligase 1 (Rli1) grow normally on *E. coli *laboratory strains, but are restricted on some *E. coli *hospital strains. The restrictive hosts are referred to as *prr*^+ ^for T4 *pnk*^- ^or *rli*^- ^mutants. T4 intergenic suppressors of the restriction of *pnk *or *rli *mutants on *prr*^*+ *^hosts define the T4 *stp *locus (see early reports cited [23,110]). The system of growth restriction results from activation of a host anticodon RNase (ACNase), PrrC, by the phage-encoded Stp protein. The bacterial PrrC RNase cuts within the tRNA^Lys ^anticodon loop, upstream of the wobble nucleotide and causes the arrest of phage protein synthesis and phage growth. T4 has evolved a tRNA repair mechanism to escape this restriction by way of the phage-induced polynucleotide kinase - 3' phosphatase (*pnk *gene), which converts the tRNA 5'-hydroxyl and 3'-phosphate termini left by PrrC, into 5'-phosphate and 3'-hydroxyl ends. Subsequently, the T4 RNA ligase 1 rejoins the tRNA ends. Stp, Pnk and Rnl1 are all under the delayed early mode of expression [23], meaning that restoration of the cleaved tRNA^Lys ^takes place early during infection. The *E. coli prrC *gene is located within a group of genes that encode type Ic restriction-modification (R-M) proteins, *Eco*prrI, in the order *hsdM-hsdS-prrC-hsdR (*or *prrABCD)*. The Hsd enzymes are assembled in a multimeric complex, HsdR_2_M_2_S [23,110-112].

#### Stp alleviates type Ic restriction and activates the tRNA^Lys ^ACNase

Although only 26 residues long, the Stp polypeptide is necessary and sufficient to elicit the tRNA^Lys ^ACNase activity and mutations in the *stp *gene abolish activation of the ACNase. Expression of Stp protein from a plasmid also elicits ACNase activity in an uninfected *prr*^*+ *^strain [113].

Stp alleviates *Eco*prrI-mediated DNA restriction, indicating that this protein targets the *Eco*prrI complex rather than just PrrC directly. Several observations support this explanation: a) Growth of the lambdoid phage, HK022, propagated on *prr*^*0 *^cells, is heavily restricted upon plating on a *prr*^*+ *^strain; b) Expression of Stp from a plasmid in *prr*^*+ *^cells alleviates this restriction; c) *prr*^*+ *^cells do not restrict growth of phage HK022 prepared on a *prr*^*+ *^host expressing Stp. This strongly suggests that Stp inhibits *Eco*prrI restriction enzyme but does not affect the modification activity. Also, the fact that *Eco*R124I, another type Ic R-M system that does not include an ACNase, is inhibited by Stp, strongly supports the above conclusion. Stp is specific for type Ic R-M systems; it has no effect on the type Ia R-M systems, *Eco*KI and *Eco*BI [113].

The N-proximal 18 amino acids of Stp protein are probably involved in the interaction with *Eco*prrI since a number of missense *stp *mutants deficient in ACNase activation have been detected among revertants of T4 *pnk*^- ^or *rli*^- ^mutants that are able to grow on the *E. coli prr*^*+ *^host. The majority of these suppressors cluster between residues 4 and 14 in the N-terminal part of the Stp polypeptide. In contrast, a deletion of 8 codons from the C-terminus only moderately decrease the two activities of Stp. Alignment of Stp sequences from eight T4-related phages with that of T4 reveals an almost absolute conservation of the 18 N-terminal residues, whereas polymorphism is evident in the remainder of the polypeptide. In most cases, the amino acids important for ACNase activation are also implicated in *Eco*prrI inhibition, suggesting some shared features [113]. We direct the reader to the primary literature by Kaufmann and colleagues [113] that hypothesizes on the evolutionary history of Stp in counteracting host DNA restriction enzymes, while also activating host ACNase.

#### Interaction of PrrC with EcoprrI and mechanism of ACNase activation

It appears that PrrC is maintained in a latent, inactive form, due to its association with the *Eco*prrI proteins. Antibodies against the closely related *Eco*R124I R-M system co-immunoprecipitate the PrrC protein. Conversely, antiserum against PrrC precipitates the HsdR (PrrD) protein [114,115].

Activation of latent ACNase in *prr*^*+ *^cell extracts requires both Stp and GTP and is likely accompanied by GTP hydrolysis since addition of the non-hydrolysable analogue, GTPγS, is inhibitory. DNA is another positive effector of ACNase. Indeed, if the cell extract is treated with DNase I, activation by Stp is abolished. The activating DNA must carry cleavable (unmodified) *Eco*prrI restriction sites to be effective in ACNase activation. This led to the proposal that Stp activates the latent ACNase when its *Eco*prrI partner is tethered to *Eco*prrI DNA substrates [114-116].

Induction of *prrC *from a multicopy plasmid elicits ACNase activity in uninfected *E. coli *cells or in cultured mammalian cells [116,117]. This occurs in the absence of any other *prr *genes. In *E. coli*, this core ACNase is highly labile (t_1/2 _< 1 minute at 30°C) while the ACNase found in extracts of *prr*^*+ *^cells is rather stable, indicating that the association with the Hsd proteins stabilizes PrrC [115]. In crude cell extracts as well as with a partially purified leaky mutant form of PrrC (more stable than the wild-type enzyme; see [22]) core ACNase is not affected by Stp and is indifferent to the presence of DNA. This suggests that the role of these two effectors is to alleviate the Hsd masking effect on PrrC [116]. dTTP and other pyrimidine nucleotides, but not GTP or ATP, stimulate core ACNase activity at physiological concentrations, most probably by stabilizing the protein. ATP, GTP and dTTP bind to the NTP-binding domain of PrrC (see below) [22,116]. Unexpectedly, GTP is inhibitory. The reason why core ACNase does not respond to GTP like the holoenzyme is unclear. Although this nucleotide binds PrrC (see below), it is possible that the GTPase catalytic site becomes active only when PrrC is associated with the Hsd component. However, it must be noted that the purified PrrC used in this study bears a leaky mutation, D222E, which confers a higher stability to the protein and permits its purification. Unfortunately, this mutation lies in the Walker B motif, which might affect the GTPase activity [22,116].

Several pyrimidine nucleotides are able to activate the latent ACNase *in the absence *of Stp, but at concentrations far above those required to protect the core ACNase or to UV-crosslink with PrrC (see below). dTTP is the most potent of them [116]. Like for Stp, the activation by dTTP requires GTP hydrolysis and *Eco*prrI DNA substrate. However, unlike Stp, which targets the Hsd complex, dTTP targets PrrC directly. The physiological meaning of this alternative mode of activation is not clear. It has been interpreted to mean that ACNase may be mobilized under cellular stress conditions not related to T4 infection. An alternative, but not exclusive, model assumes that dTTP is an obligatory co-activator working in concert with Stp. Because dTTP binds PrrC with high affinity, trace amounts of this nucleotide in crude extracts would be sufficient to allow latent ACNase activation upon Stp addition. Excess dTTP would by-pass the requirement for Stp [22].

#### PrrC structure, domain organization and distribution

**Figure 6:**
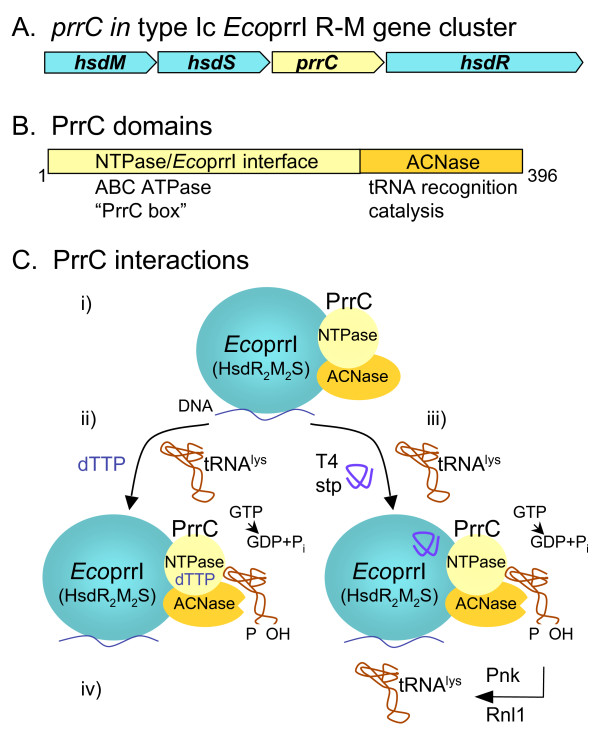
**PrrC tRNA anticodon nuclease and T4 exclusion system**. **A) **The *E. coli hsd *gene cluster includes *prrC*. **B) **PrrC has N-terminal two thirds NTPase and *Eco*prrI interaction domains and, starting at residue 265, C-terminal tRNA recognition and ribonuclease (ACNase) catalytic domains. **C) **Current model for tRNA cleavage and T4 exclusion. i) PrrC, minimally as a head-to-tail dimer (tetramer and hexamer oligomers are possible) associates with *Eco*prrI on DNA, as an inactive, latent endoribonuclease. ii) In one of two allosteric activation mechanisms, *Eco*prrI-PrrC-DNA complex binds increased levels of dTTP and with GTP hydrolysis activated ACNase cleaves the anticodon of tRNA^Lys^. iii) During infection, the small, T4-encoded polypeptide stp binds *Eco*prrI activating tRNA^Lys ^ACNase. iv) T4 repairs the cleaved tRNA at the 2',3'cyclic phosphate and 5' OH using polynucleotide kinase (Pnk) and RNA ligase (Rnl1). *Figure adapted from the publications of Kaufmann and colleagues *[111,114,116,119].

The N-proximal two-thirds of the PrrC protein (ca. 265 residues out of 396) harbors a nucleotide-binding site and is thought to mediate activation of the latent ACNase (Figure 6). It features motifs that resemble those found in typical ABC-transporter ATPases: a somewhat degenerated ABC signature motif, Walker A (phosphate-loop) and Walker B motifs and an H-motif that contains a highly conserved His (the linchpin His) [22]. Mutations in the universally conserved residues of the Walker A motif of PrrC abolish ACNase activity [116]. ATP, GTP and dTTP bind PrrC to this region, as a mutation lying immediately upstream of the ABC signature severely decreases the ability of the protein to UV-crosslink with all three nucleotides. Because dTTP activates ACNase by targeting PrrC directly and requires GTP hydrolysis, the binding sites for the two nucleotides are likely different in PrrC oligomer. GTP and ATP likely share the same site. The interaction of dTTP with PrrC departs from that of the two other nucleotides in several respects. Mutations in the N-proximal Walker A motif, in the ABC signature sequence, or in the linchpin His do not affect the binding of ATP or GTP while they abolish dTTP binding to PrrC. Also, dTTP affinity to PrrC is three orders of magnitude higher than that of the two other nucleotides. Furthermore, a mild heat inactivation of ACNase has little consequence on ATP or GTP binding but abolishes dTTP binding. This suggests that the dTTP binding site is distinct and is sensitive to small changes in PrrC structure [22,116].

The C-terminal third of PrrC is implicated in tRNA recognition and catalysis. Several missense mutations affecting ACNase activity are located in close proximity here (Figure 6). These were selected as mutations conferring the ability to survive the lethal overproduction of PrrC [118]. Because most of these substitutions are clustered in a short sequence highly conserved in a subset of the known PrrC homologues (residues 287 to 303) [22,118,119], the behavior of an 11-residue peptide (residues 284 to 294) was examined for RNA substrate interactions. This peptide forms UV-induced crosslinks with tRNA^Lys ^anticodon stem-loop analogs and inhibits the ACNase activity of PrrC. Introducing certain substitutions in the peptide that are known to inactivate full-length PrrC, or shortening it by one amino acid from either end, leads to strong decrease in its ability to inhibit ACNase and to UV-crosslink with the anticodon stem-loop substrates [119]. Thus, this sequence is likely a part of the PrrC protein that interacts with the tRNA. In addition, substitutions in Arg320, Glu324 and His356 that are 100% conserved in all known PrrC homologs and suspected to participate in the acid-base catalytic mechanism, completely abolish ACNase activity. Null mutations in Arg320 and Glu324 can be rescued chemically by small molecules, indicating that the ACNase deficiency does not arise from a change in the structure of the protein, but rather from the lack of the correct amino acid side chain. This is compatible with the notion that at least two of the three conserved residues are implicated in catalysis [22].

Orthologues of *prrC *were found in 19 distantly related bacteria, all linked to genes for type Ic R-M enzymes. All of the orthologue proteins share in their N-terminal domains the NTP-binding site and a sequence of 15 residues called the "PrrC box". Also, their C-terminal domains contain the catalytic amino acid triad mentioned above. Thus, the PrrC proteins form a family whose members are strongly suspected not only to possess anticodon nuclease activity (as shown to be the case for those encoded by *Haemophilus influenzae *and *Streptococcus mutans *[120]), but also to be regulated like *E. coli *PrrC. However, their substrates may vary since the sequence involved in tRNA recognition varies among the PrrC proteins [22,119].

ACNase activity co-elutes from a gel filtration column with a homo-oligomer of ca. 200 kDa, suggesting that active PrrC could be a tetramer. Glutaraldehyde protein-protein crosslinking experiments confirm this, as mostly dimers and tetramers are produced [22]. Klaiman *et al*. [119] showed evidence suggesting that the C-terminal region of PrrC, involved in tRNA recognition, interacts with the substrate as a parallel dimer. Thus, while the N-terminal domain of PrrC is expected to associate in a head-to-tail dimer, by analogy with known structures of ABC transporter ATPases, the C-terminal region seems to dimerize in opposite orientation. To account for this situation, Klaiman et al. [119] proposed a model in which the PrrC subunits are associated in a unique tetramer conformation. Clearly, additional structural studies are necessary to elucidate the oligomeric structure of PrrC.

#### ACNase specificity

Kaufmann and colleagues have shown that the tRNA^Lys ^anticodon stem-loop region plays a prominent role in PrrC recognition: (a) PrrC, when overproduced in cells, cleaves other tRNAs in addition to tRNA^Lys^. The anticodon sequences of all these secondary tRNA substrates share sequence similarities with that of tRNA^Lys ^[118]. (b) Expression of PrrC in human HeLa cells elicits cleavage of intracellular tRNA^Lys3 ^that shares with the *E. coli *tRNA^Lys ^the same anticodon loop sequence [117]. (c) Most mutations in the tRNA^Lys ^anticodon sequence make the resulting tRNAs very poor substrates for ACNase. One of them, however, (U35 -> C leading to UCU anticodon) leads to relaxed site specificity as new cleavages occur upstream and downstream of the usual cleavage site [121]. (d) A chimeric, unmodified, tRNA^Arg1 ^carrying the UUU lysine anticodon instead of its own anticodon, is as efficiently cleaved as the unmodified tRNA^Lys ^[121]. (e) PrrC quite efficiently cleaves a fragment of the tRNA^Lys ^encompassing only the anticodon loop and the first 5 base pairs of the associated stem (17 nucleotides altogether) [122]. (f) Cleavage of tRNA^Lys ^that lacks either of the two modifications of the uridine wobble base (2-thio- and 5-methylaminomethyl) is severely affected. Interestingly, three substitutions of PrrC Asp287 (D287Q, D287H and D287N), known to reduce the efficiency of cleavage of normally modified *E. coli *tRNA^Lys^, reverse the negative effect of the hypomodifications of the wobble base. This strongly supports the notion that Asp287 directly contacts the modified wobble base. Experiments carried out with the anticodon stem-loop (17-mer) as substrates reinforce this conclusion. Indeed, Jiang *et al*. [122] showed that the wobble base modification present in the anticodon stem-loop derived from mammalian tRNA^Lys3 ^(5-methoxycarbonyl-2-thiouridine instead of 5-methylaminomethyl-2-thiouridine) is inhibitory to ACNase activity. However, D287H PrrC, poorly active on the fully modified *E. coli *anticodon stem-loop counterpart, overcomes this inhibitory effect [121,122]. (g) The influence of the stem stability and of the three different modifications in these anticodon stem-loop structures was examined in great detail. The picture that emerges is the following. A stable stem is inhibitory to ACNase activity. Some breathing of the duplex seems necessary, possibly to facilitate conformational changes of the tRNA upon interaction with PrrC. Also, PrrC seems to favor base modifications that help stack the anticodon nucleotides into an A-RNA conformation [122]. Thus, three elements are recognized by PrrC: the anticodon sequence, the base modifications and base-pairing of the stem.

Although the anticodon stem-loop region of tRNA^Lys ^is the predominant element of PrrC specificity, other sequence and/or structural elements of tRNA^Lys ^seem to be involved. This is indicated by the fact that chimeric tRNAs, other than the tRNA^Arg1^, carrying the lysine anticodon, are not substrates for PrrC. Also, any substitution of the discriminator nucleotide (A73) of the tRNA^Lys^, a major identity element of LysRS that lies in the acceptor arm, reduces, though moderately, the ACNase cleavage efficiency. Furthermore, trimming the 3'-terminal ACCA overhang nucleotides has little effect on ACNase activity but relaxes the cleavage site specificity in a manner similar to the U35 -> C mutation [121]. These data suggest additional interactions between PrrC and the acceptor region of tRNA^Lys^.

#### Gathering the data into a model

Taken together, the above data suggest the following cascade of events. A few minutes after infection of *prr*^*+ *^*E. coli *cells, the T4-encoded Stp polypeptide binds the bacterial *Eco*prrI component and inhibits its DNA restriction activity (Figure 6). This modifies the *Eco*prrI/PrrC interaction, inducing a change in PrrC conformation that unmasks ACNase activity. This process requires GTP hydrolysis. dTTP, bound to PrrC, is a co-activator with Stp. Its role could be to stabilize PrrC that would otherwise be labile in its activated conformation. The tRNA^Lys ^anticodon is then bound and cleaved by the respective ACNase regions. But the phage provides the healing (Pnk) and sealing (Rnl1) enzymes required to restore the affected tRNA, allowing the phage to escape the cellular defense. The phage exclusion mechanism depicted here and the way the phage wards off this cellular defense revealed an intimate physiological link between restriction-modification regulation and translational activity. The distribution of PrrC homologs in unrelated bacteria and their systematic link with type Ic R-M systems, suggest that the PrrC proteins have a cellular function not related to phage infection, possibly to disable protein synthesis under conditions of stress that affect activity of type I DNA restriction endonucleases [22,111,120].

#### Cellular RloC proteins

Using a bioinformatical approach, Davidov *et al*. [120] recently found a new class of PrrC homologs called RloC (restriction linked orf). RloC proteins are widespread in bacteria, although they are not present in *E. coli *and only one was found in Archaea and none in Eukarya. Genes for some of these proteins were first characterized as linked to genes for type I or III R-M enzymes in *Campylobacter jejuni *[123] however, now only a minority of the *rloC *genes map to R-M loci. RloC orthologues share with *E. coli *PrrC the presence of ATPase motifs in the N-termini and the amino acid triad thought to constitute the catalytic site in the C-termini. This structural homology is accompanied by a functional homology: when expressed in *E. coli*, RloC from the thermophilic *Geobacillus kaustophilus *exhibits "ACNase" activity. Also, alanine substitutions of the three amino acids of the triad abolish RloC ACNase. However, RloC differs from PrrC in several respects: (1) RloC substrate is still uncertain but it is not tRNA^Lys^; (2) RloC ACNase actually *excises *the wobble nucleotide rather than just cleaves upstream; and (3) Like the other RloC orthologues, the *G. kaustophilus *protein is larger than PrrC because the N-terminal NTPase domain is interrupted by a large coiled-coil fragment that's similar to sequences found in proteins implicated in DNA repair. This fragment contains a typical "zinc hook" motif able to co-ordinate Zn^+2 ^ions. Mutations in the zinc-hook motif lead to increased ACNase activity and conversely, Zn^+2 ^ions are inhibitory [120].

The RloC proteins show quite interesting and new properties that lead to several questions.

a) Is the RloC-dependent ACNase normally maintained in a latent, inactive form that is activated upon phage infection?

b) Since RloC excises the wobble nucleotide, is there a phage that repairs this lesion? If not, this would be an efficient mechanism of cellular defense against phages.

c) Are there stress conditions, unrelated to phage infection, that elicit RloC ACNase activation?

d) Are the RloC proteins associated with restriction-modification proteins? If so, do they respond to the presence of DNA?

By analogy with the PrrC ACNase, Kaufmann and colleagues [120] speculate that, in addition to conferring a mechanism of phage exclusion, the RloC proteins couple DNA damage that occurs under stress conditions, to translation inactivation *via *tRNA cleavage. Their model is based on two main observations: a) some proteins containing zinc-hook/coiled-coil domains are implicated in DNA repair; and b) DNA damage leads to alleviation of type Ia and Ic restriction enzymes, a process aimed at protecting unmodified, newly synthesized DNA during the process of repair and recovery from damaged DNA. The model assumes that the RloC protein would sense DNA damage signals via its zinc-hook and would convey activation to the ACNase domain, possibly via conformation changes driven by NTP hydrolysis. Such a model requires demonstrating a link between RloC proteins and DNA.

### T4 exclusion by Gol-activated proteolysis of EF-Tu

Translation elongation is targeted in the T4 *gol-lit *phage exclusion system [23,110]. Inhibition of translation occurs when T4 gene *23 *(the major head protein) is translated during infection of *E. coli *cells that harbor the defective prophage e14. The e14 element carries the *lit *gene, which encodes a latent protease that, somewhat similar to allosteric activation of latent PrrC ACNase activity, is active on EF-Tu when the so-called *gol *region of gene *23 *is translated. Biochemical analyses of Lit/Gol/EF-Tu interactions have revealed the process by which phage exclusion occurs through proteolysis of EF-Tu.

A short 29-residue region of the gp23 polypeptide defines Gol function, but a more stable interaction with Ef-Tu appears to occur with 100 amino acids from the first ¼ of gp23. Scanning mutagenesis showed 13 residues in a 20 amino acid core region of Gol to be most important for its activity [124]. Binding of Gol to EF-Tu is required to promote Lit reactivity. By binding to domains II and III of EF-Tu, the Gol peptide promotes Lit-mediated hydrolysis of EF-Tu between Gly59 - Ile60. Binding of Gol peptide is preferential for the open EF-Tu:GDP complex, and binding itself inhibits the EF-Tu GTPase of domain I. When Gol is bound to EF-Tu, it appears that EF-Tu domain I is more accessible to Lit, leading to "substrate-assisted" or "cofactor-induced" activation of cleavage by the protease [124,125]. Lit is a zinc metallo-protease with the active site motif HEXXH of this protease class, but Gol does not contribute directly to active site residues [124-126]. Kleanthous and colleagues [124] have noted that the gp23 Gol region is the most conserved region, in the overall conserved gp23 major head protein of sequenced T4-related phages. They suggest that gp23 of these phages interacts with EF-Tu of all the respective hosts. While Gol interactions may be broadly relevant for translation and folding of this extremely abundant capsid protein, other extant prophage-encoded, Lit-type proteases may also elicit "cellular suicide" via Lit/Gol/EF-Tu proteolytic assemblies.

### Programmed translational bypassing

**Figure 7:**
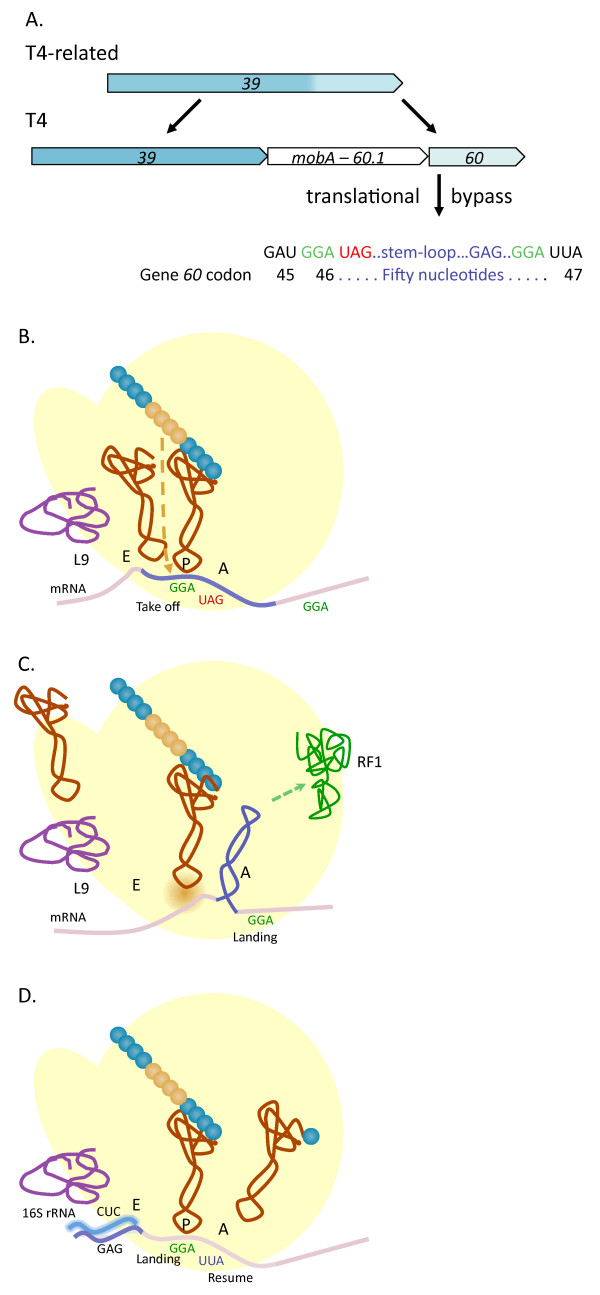
**Model for programmed translational bypassing in T4 gene *60***. **A**) In most T4-related phages, the large topoisomerase subunit (gp39) is encoded by a single gene. In T4, active site domains are interrupted by the *mobA-60.1 *coding region where the downstream ORF (gp60) contains the 50 nucleotide bypassed sequence. Elements in the mRNA that code for translational bypassing are shown. **B**) Recapitulation of the model [127] where E-, P- and A- sites of the ribosome are shown as translation enters the bypass region (blue mRNA). Nascent peptide is shown in gold and as promoting "take-off" from the green GGA codon. In (**C**) the bypass mRNA stem-loop structure occupies the A-site, precluding release factor 1 [66] from binding and inhibiting termination. Ribosomal protein L9 facilitates tRNA exit through the E-site and nascent peptide interactions prevent peptidyl-tRNA scanning. **D**) Landing site codon:anticodon pairing is shown, with entry of the resuming charged tRNA into the A-site for translating the distal region of gene *60 *mRNA. The GAG six nucleotides 5' of the landing site is shown pairing with the 16S rRNA anti-SD region to help reinitiate peptidyl-tRNA scanning and pairing at the GGA. *Figure adapted from *[127], *with kind permission of J. F. Atkins*.

Topoisomerase of phage T4 is encoded by three genes: *39*, *60 *and *52*. Most type II topoisomerases are comprised of two distinct subunits (i.e., *gyrA *and *gyrB *of DNA gyrase) that are assembled as tetrameric A_2_B_2 _enzymes. The adjacent T4 genes *39 *and *60 *are separated by 1010 nucleotides that include an apparently defective HNH homing endonuclease gene (*mobA*) and ORF *60.1 *[95] (see [127] for a recent summary). Following their respective translation, gp39 and gp60 assemble to comprise the "gyrB-like" large, ATP-hydrolyzing subunit of the T4 topoisomerase. In all other T4-related phages sequenced to date, this subunit is encoded by a single open reading frame that is typically annotated as gene *39 *(Figure 7).

An interesting, post-transcriptional feature in this region of the T4 genome that has received considerable attention is the presence of a 50 nucleotide "intervening sequence" in the 5' coding region of gene *60 *that is transcribed into mRNA but is not translated into gp60. The ribosome "hops" or "bypasses" the extra 50 bases in the mRNA to produce the gp60 polypeptide in a process termed programmed translational bypassing [128,129]. In all other T4-related phages, not only are genes *39 *and *60 *joined as a single gene (they lack the *mobA - 60.1 *insertion), but none appears to have the intervening gap nucleotides that would suggest programmed translational bypassing. The process appears to be unique to T4 gene *60*, but its study has shed new light on the mechanisms of translation. Atkins, Gesteland and colleagues at the University of Utah have studied many of the features that promote programmed translational bypassing by *E. coli *ribosomes on this unique T4 mRNA; the reader is urged to read further details through their primary research articles and reviews on the topic [129-131]. It is important to emphasize that experiments elucidating processes affecting gene *60 *translational bypassing have provided new insights to the general mechanisms of mRNA decoding, including the roles of mRNA sequence and structure, peptidyl-tRNA interactions within the ribosome, occupancy of ribosome decoding sites, and many other features of translating ribosomes.

Figure 7 summarizes the major components of the T4 programmed translational bypass in gene *60 *transcripts, as elaborated by the Utah group. Some of principal features include: a domain of the nascent gp60 polypeptide preceding the hop, the glycine 46 codon GGA at the end of the initial ORF (the "take-off site"), a UGA stop codon in the gap right after the GGA take-off codon, a stem-loop mRNA structure with a stabilizing tetraloop that is formed by gap RNA nucleotides, the peptidyl-tRNA_2_^Gly ^occupying the P site of the ribosome, rRNA:mRNA interactions during scanning of the gap by the ribosome, the L9 subunit of the ribosome, and the 50 nucleotide distal GGA codon after the gap nucleotides (the "landing site") [127,132-138]. Protein fusions, mass spectrometry, targeted mutations and a number of analyses have combined to address the roles of each component, leading to an approximately 50% efficiency of ribosomes bypassing the intervening 50 nucleotides to land correctly at the downstream GGA codon. Translation then resumes as the next UUA codon and cognate tRNA enter the A site. Again, each of these features shed new light not only on mechanisms of translational bypassing and aspects of "re-programming" the basic genetic code, but also on the dynamics and numerous interactions occurring in all translating ribosomes. It will be interesting to see whether instances of programmed translational bypassing occur in other genes of the many T4-related bacteriophages.

### ADP-ribosyltransferases in post-transcriptional control

T4 encodes three enzymes that covalently modify proteins via ADP-ribosylation during the infection cycle: Alt, ModA and ModB. Alt is injected with phage DNA to immediately initiate ADP-ribosylation of one of the α-subunits (at arginine 265) of RNA polymerase, and by about 4 minutes post-infection newly synthesized ModA completes modification of both α-subunits at the same arginine. The biochemistry of T4-directed ADP-ribosylation of RNA polymerase and its impact on phage promoter selection have been reviewed [2,139].

Other *E. coli *proteins have been recognized as undergoing ADP-ribosylation during T4 infection, which primarily appears to be due to the activity of Alt and ModB. Alt ADP-ribosylates β, β' and σ subunits of RNA polymerase and also other host proteins. The modifications also include proteins of the translation apparatus, as shown by Ruger and colleagues using the purified proteins [140]. An *in vitro *system incubated with total *E. coli *proteins (cell extract), purified Alt or ModB, and ^32^P[NAD^+^] showed with mass spectrometry that ADP-ribosylation occurred on as many as 27 proteins by Alt and on approximately 8 proteins by ModB [141]. For Alt, these included EF-Tu, trigger factor, prolyl-tRNA synthetase and GroEL that are known to have important roles in translation or protein folding. ModB also ADP-ribosylates EF-Tu and trigger factor, as well as ribosomal protein S1 [142]. For trigger factor (a chaperone of newly translated proteins), arginine 45 is ADP-ribosylated by ModB, but not by Alt, which must target a different, as yet unidentified amino acid [141]. Arg45 lies in the Phe44-Arg45-Lys46 domain that interacts with ribosomal protein L23, and thus might affect ribosome conformation and translation, as certainly would modifications to EF-Tu and S1. Early studies noted rapid & immediate shut-off of host mRNA translation during T4 infection [143], but no clear mechanism has been elucidated. The identification of pivotal proteins in the translation apparatus as targets of T4 ADP-ribosyltransferases, together with the observed delivery of Alt into the cell with the injected DNA and the lethality to the cell of over-expressed ModB [142], suggest that mechanistic studies into the impact of these T4 enzymes on translation of host and phage mRNAs is warranted.

## Conclusions

Although post-transcriptional control in T4 development and gene expression has been appreciated and studied for decades, many of the molecular details, especially for specific RNA-protein interactions, have yet to be resolved. For most, crystal or solution structures of bound mRNA-repressor or RNA-nuclease complexes would significantly advance our understanding of complex formation and substrate interactions in catalysis. While clearly germane to T4 and the large diversity of T4-related bacteriophages in the biosphere, continued study of post-transcriptional processes directed by these phages will provide new advances in the biochemistry pertinent to all cellular systems. Undoubtedly new anti-virals and anti-microbials targeting these and related systems in pathogens can be anticipated.

## Competing interests

The authors declare that they have no competing interests.

## Authors' contributions

MU and ESM wrote the manuscript and approved the final version.
